# Progressive Changes in CXCR4 Expression That Define Thymocyte Positive Selection Are Dispensable For Both Innate and Conventional αβT-cell Development

**DOI:** 10.1038/s41598-017-05182-7

**Published:** 2017-07-11

**Authors:** Beth Lucas, Andrea J. White, Sonia M. Parnell, Peter M. Henley, William E. Jenkinson, Graham Anderson

**Affiliations:** Institute for Immunology and Immunotherapy, College of Medical and Dental Sciences, Medical School, University of Birmingham, Birmingham, B15 2TT England

## Abstract

The ordered migration of immature thymocytes through thymic microenvironments generates both adaptive MHC restricted αβT-cells and innate CD1d-restricted iNKT-cells. While several chemokine receptors and ligands control multiple stages of this process, their involvement during early thymocyte development often precludes direct analysis of potential roles during later developmental stages. For example, because of early lethality of CXCR4^−/−^ mice, and stage-specific requirements for CXCR4 in thymus colonisation and pre-TCR mediated selection, its role in thymic positive selection is unclear. Here we have examined CXCR4-CXCL12 interactions during the maturation of CD4^+^CD8^+^ thymocytes, including downstream stages of iNKT and αβT-cell development. We show CXCL12 expression is a common feature of cortical thymic epithelial cells, indicating widespread availability throughout the cortex. Moreover, CXCR4 expression by CD4^+^CD8^+^ pre-selection thymocytes is progressively downregulated following both MHC and CD1d-restricted thymic selection events. However, using CD4^Cre^-mediated deletion to bypass its involvement in CD4^−^CD8^−^ thymocyte development, we show CXCR4 is dispensable for the maintenance and intrathymic positioning of CD4^+^CD8^+^ thymocytes, and their ability to generate mature αβT-cells and CD1d-restricted iNKT-cells. Collectively, our data define dynamic changes in CXCR4 expression as a marker for intrathymic selection events, and show its role in T-cell development is restricted to pre-CD4^+^CD8^+^ stages.

## Introduction

In the immune system of vertebrates, the thymus controls the development of αβT-cells that play multiple and essential roles in immune responses^1^. In the adult thymus, intrathymic microenvironments are heterogeneous, with the cortex and medulla being formed from a range of non-hemopoietic stroma including cortical and medullary thymic epithelial cells (cTEC and mTEC respectively), mesenchymal and endothelial cells^2, 3^. Importantly, these distinct thymic areas house developing thymocytes at differing stages of maturation, enabling specific stromal cells to provide essential signals for thymocyte development^4, 5^. For example, immature CD4^−^CD8^−^ double negative (DN) T-cell precursors reside within subcapsular and cortical regions, CD4^+^CD8^+^ double positive (DP) thymocytes are limited to the cortex, and mature CD4^+^CD8^−^ and CD4^−^CD8^+^ single positive (SP) thymocytes locate to the medulla prior to their exit from the thymus. This intrathymic positioning of thymocytes occurs as a result of their step-wise migration during development. Thus, entry of the most immature lymphoid precursors takes place at the cortico-medullary junction^6, 7^ which is followed by migration of DN thymocytes towards the subcapsular region^6, 8^, with DP thymocytes then filling the cortex as they traverse back through the thymus towards the medulla, where they arrive as SP cells^9, 10^. Consequently, in current models of adult thymus function, αβT-cell development depends upon a complex migratory pathway for developing thymocytes.

Multiple chemokine receptors and their ligands influence thymocyte migration processes, and so ensure access to appropriate thymic microenvironments. CXCR4, CCR7 and CCR9 are all expressed by T-lymphoid progenitors and contribute to their entry into the thymus^11–15^. Interestingly, chemokine receptor expression is highly dynamic during thymocyte development, suggesting specific roles at particular developmental stages. For instance, while downregulation of CCR7 occurs during DN stages so that pre-selection DP thymocytes are CCR7^−^, positive selection creates newly generated CCR7^+^CD4^+^ and CCR7^+^CD8^+^ SP thymocytes that enter the medulla for tolerance induction^9, 16–19^. In contrast to CCR7, DN and DP thymocytes express both CCR9^20–24^ and CXCR4^20, 25–30^ suggesting additional roles for these receptors during multiple developmental stages. For example, DP thymocytes demonstrate chemotactic responsiveness to CXCL12^31^ and *in vitro* treatment of mouse thymic slices with the CXCR4 antagonist AMD3100 results in the mis-localisation of human DP thymocytes to the medulla^26^, suggesting it may act as a retention factor that maintains DP thymocyte positioning in the cortex. Moreover, *in vitro* assays also suggest that CXCL12 acts as a chemorepellent during the exit of mature SP cells from the thymus, a processes termed chemofugetaxis^29, 32^. Despite these observations, genetic analysis of the role of CXCR4-CXCL12 during steady state T-cell development in the adult thymus is lacking, which is due at least partly to the embryonic and postnatal lethality caused by CXCR4 and CXCL12 deficiency^33–36^. Interestingly, several studies have employed Cre-mediated deletion of CXCR4 in DN thymocytes, with p56lck^Cre^CXCR4^fl/fl^ mice revealing a role in early T-cell development^25, 28, 37^. Importantly, the role of CXCR4 appears to extend beyond the positional regulation of immature thymocytes, with CXCR4 exerting a critical impact on DN thymocyte proliferation and survival during β-selection via co-stimulatory interplay with the pre-TCR^28^. However, these p56lck^Cre^CXCR4^fl/fl^ models preclude analysis of later developmental stages due to impaired DN to DP transition. When taken together with conflicting reports on the intrathymic distribution of CXCL12^20, 25, 27, 28, 38, 39^, the *in vivo* requirement for CXCR4-CXCL12 interactions downstream of DP thymocytes remains unclear.

Here, we have examined the intrathymic expression of CXCR4 and CXCL12, and have generated CD4^Cre^CXCR4^fl/fl^ mice to bypass the requirement for CXCR4 in DN thymocytes, allowing direct analysis of the role of CXCR4 in DP thymocytes and subsequent downstream developmental stages. Using CXCL12^dsRed^ reporter mice, we show the majority of cTEC are CXCL12^dsRed+^, resulting in its widespread expression throughout the cortex. Thus, CXCL12 is available to most if not all DP thymocytes, suggesting that it may be an important regulator of cortical thymocytes, including DP positioning, and positive selection of conventional αβTCR^+^ and CD1d-restricted iNKT-cells. In addition, we show that while both CCR9 and CXCR4 are uniformly expressed by pre-selection DP thymocytes, they show distinct differences in their patterns of expression following positive selection. Thus, unlike CCR9, pre-selection DP CD69^−^CXCR4^hi^ cells undergo rapid CXCR4 downregulation following positive selection initiation to generate DP CD69^+^CXCR4^low^ cells, followed by CD4^+^ and CD8P^+^ SP thymocytes that lack detectable CXCR4 expression. Despite this significant shift in chemokine receptor expression and the maintenance of CXCR4 expression by positive selection intermediates, we show that in both steady state and competitive conditions, conventional and CD1d-restricted αβT-cell development occurs normally in CD4^Cre^CXCR4^fl/fl^ mice. Thus, while differing levels of CXCR4 expression by DP thymocytes serve as an indicator of cells undergoing TCR-mediated thymic selection, we show that unlike for the DN compartment, CXCR4 is not required for survival/homeostasis and downstream development of the pre-selection DP compartment.

## Results

### Widespread Expression of CXCL12 In The Thymic Cortex

**Figure 1:**
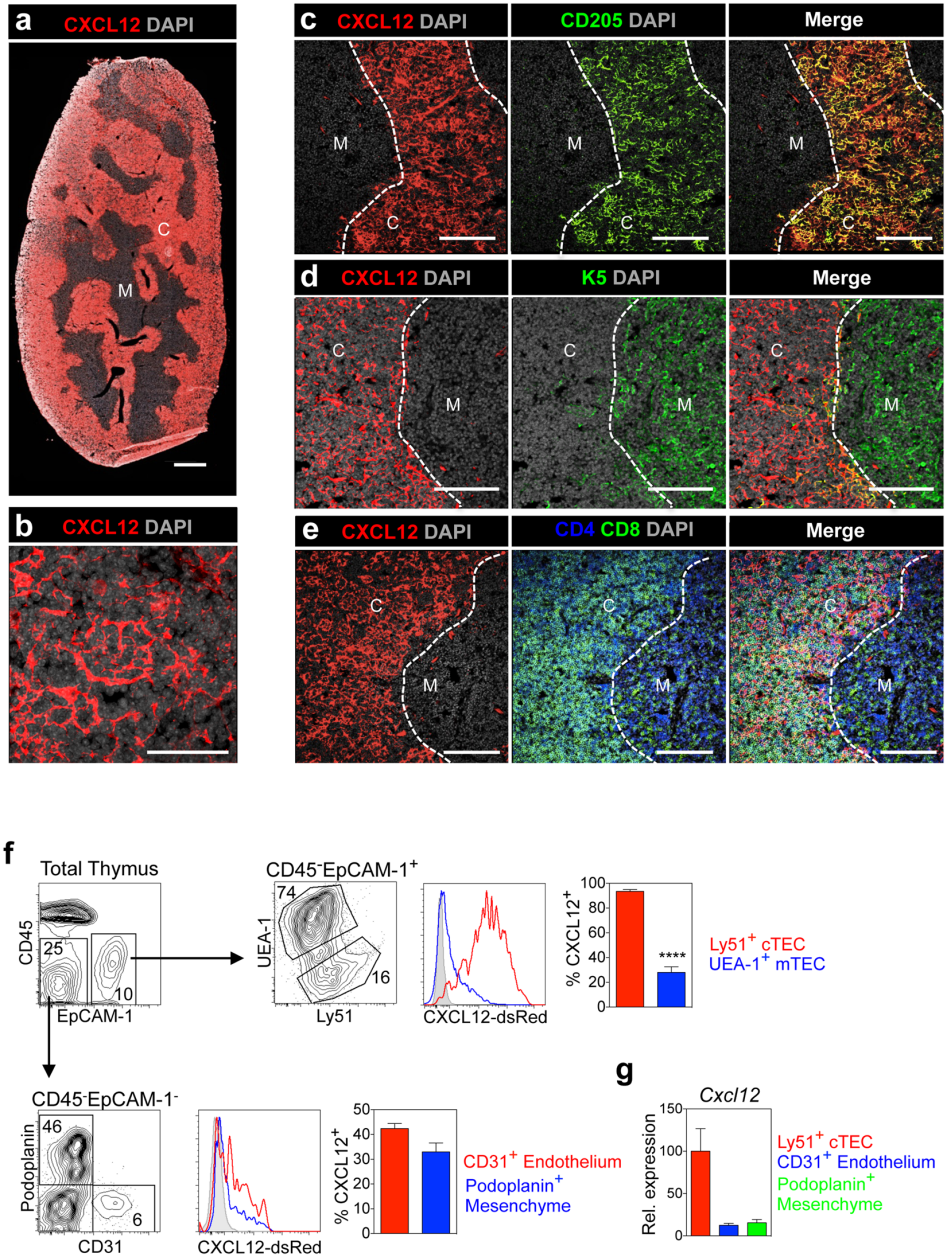
CXCL12 Is Expressed Throughout The Thymic Cortex. Confocal images showing (**a)** expression of CXCL12^dsRed^ in the thymus, scale bar represents 500 μm (**b)** reticular morphology of CXCL12^dsRed^ expressing cells, scale bar represents 50 μm (**c**) co-localisation of CXCL12^dsRed+^ cells and CD205^+^ cTEC, (**d)** absence of CXCL12^dsRed^ expression by K5^+^ mTEC, (**e)** restriction of CXCL12^dsRed+^ cells to areas of the thymus containing CD4^+^CD8^+^ DP thymocytes. ‘C’ denotes cortex and ‘M’ denotes medulla, confocal images are representative of at least three mice. Scale bars in b-d represent 100 μm. (**f)** Representative FACS analysis and quantitation of CXCL12^dsRed^ expression by UEA-1^+^EpCAM-1^+^ mTEC and Ly51^+^EpCAM-1^+^ cTEC, CD31^+^ endothelium and podoplanin^+^ mesenchyme. n > 5, unpaired Student’s *t* test ****p < 0.0001. (**g)** Purified thymic stromal cells from WT mice were analyzed by qPCR for *Cxcl12* mRNA, normalized to β-actin (mean ± SEM). qPCR data is representative of at least two independent biological experiments. Error bars represent SEM.

The ability of cTEC to regulate early T-cell development maps to their expression of key genes including β5t, DLL4 and CXCL12. However, while β5t and DLL4 are expressed by most if not all cTEC^40, 41^, the precise intrathymic distribution of CXCL12 producing cells is not clear^20, 25, 27, 28, 42^. As a first step in examining the role of CXCL12-CXCR4 interactions during DP thymocyte development, we determined the intrathymic expression pattern of CXCL12 using CXCL12^dsRed^ reporter mice, in which the fluorescent protein dsRed is knocked into the CXCL12 locus^43^. Confocal analysis of thymus sections from adult mice revealed widespread expression of CXCL12^dsRed^ within the cortex, with medullary areas notably lacking CXCL12^dsRed+^ cells (Fig. 1a). Higher magnification images within cortical areas displayed a reticular network of CXCL12^dsRed+^ cells with morphological features of cTEC (Fig. 1b). Additional staining with antibodies to the cTEC marker CD205 showed co-expression of CD205 and CXCL12 in cortical areas (Fig. 1c), while Keratin-5^+^ medullary areas were CXCL12^dsRed−^ (Fig. 1d). Consistent with this cortical pattern of expression, CXCL12^dsRed+^ cells were positioned within areas of the thymus containing DP thymocytes (Fig. 1e).

To further examine the nature of CXCL12^+^ cells within the thymus, we digested CXCL12^dsRed^ thymi and performed flow cytometry using a panel of stromal antibodies to allow detection of EpCAM-1^+^UEA-1^+^ mTEC, EpCAM-1^+^Ly51^+^ cTEC, EpCAM-1^−^podoplanin^+^ mesenchyme, and EpCAM-1^−^CD31^+^ endothelium. Consistent with confocal analysis of tissue sections, almost all cTEC expressed high levels of CXCL12^dsRed^ (Fig. 1f). Interestingly, and in addition to TEC, CXCL12^dsRed^ expression was also detected within both mesenchymal and endothelial cells (Fig. 1f). Flow cytometric analysis of CXCL12^dsRed^ expression paralleled qPCR analysis of *Cxcl12* mRNA expression in purified cTEC, endothelium and mesenchyme isolated from adult mouse thymus, with highest *Cxcl12* mRNA levels detectable in cTEC (Fig. 1g). Thus, use of fluorescent reporter knock-in mice shows that CXCL12 is widespread throughout the thymic cortex as a result of its predominant expression by the majority of cTEC.

### Progressive Changes In CXCR4 Follow Positive Selection of Conventional αβT-cells

**Figure 2:**
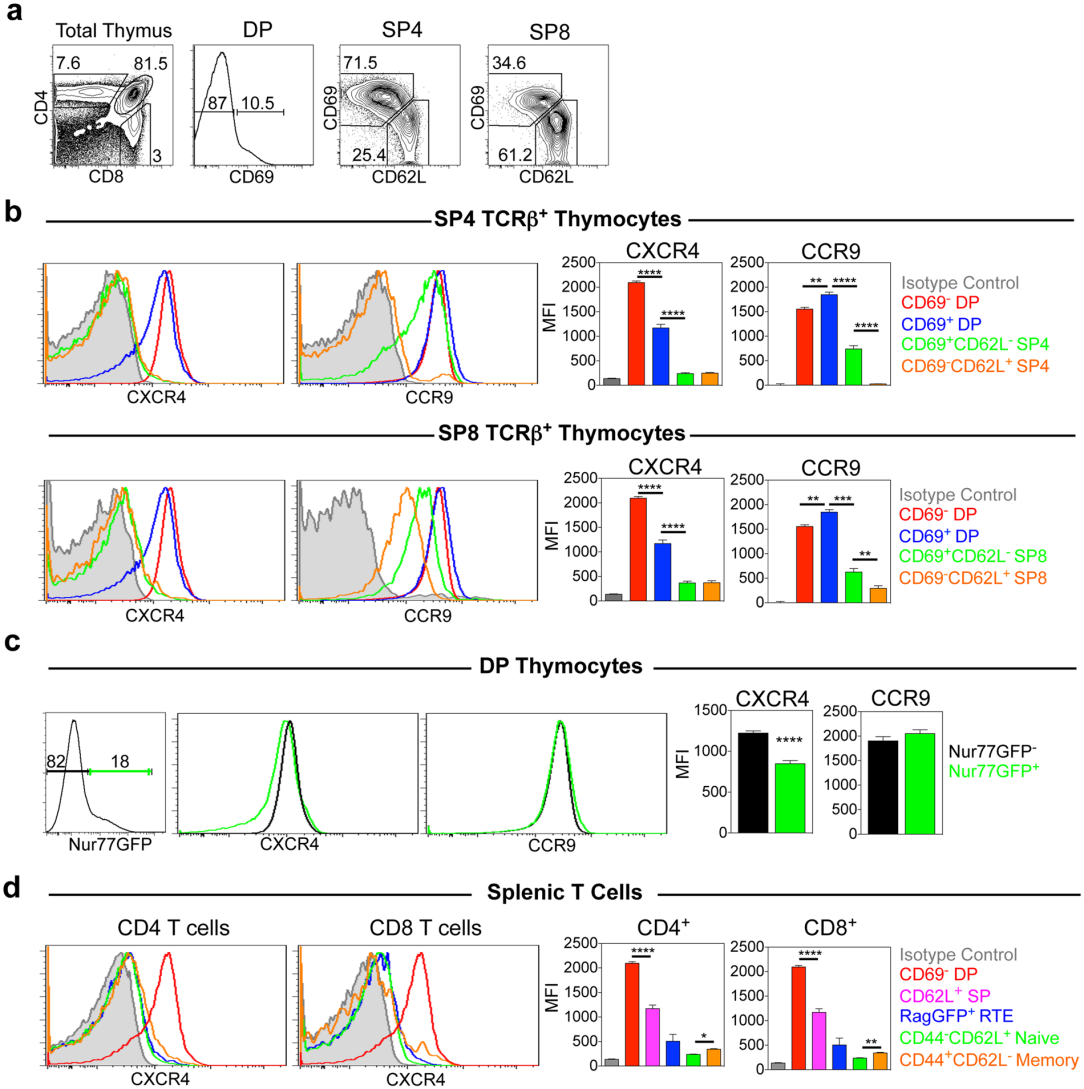
Positive Selection Initiation Involves Progressive CXCR4 Downregulation In DP Thymocytes. (**a**) Identification of CD69^−^ DP, CD69^+^ DP, CD69^+^CD62L^−^, and CD69^−^CD62L^+^ SP4 and SP8 thymocytes. (**b**) Representative FACS analysis and MFI of CXCR4 and CCR9 expression in the indicated thymocyte subsets isolated from adult WT mice. (**c**) Representative FACS analysis and MFI of CXCR4 and CCR9 expression by Nur77GFP^+^ and Nur77GFP^−^ DP thymocytes from Nur77GFP mice. (**d**) Representative FACS analysis and MFI of CXCR4 expression by splenic T cell subsets in WT Rag2GFP mice, RTE (Recent Thymic Emigrants, Rag2GFP^+^), Naïve T cells (CD62L^+^CD44^−^), Memory T cells (CD62L^−^CD44^+^). Grey histograms in b and d show isotype control staining for CXCR4 or CCR9, pre-gated on CD69^−^ DP thymocytes. Graphs in d show MFI of CXCR4 on CD62L^+^ SP thymocytes and CD69^−^ DP thymocytes for comparison. MFI analysis is calculated from one experiment, representative of at least 3 independent experiments. n > 10, unpaired Student’s *t* test *p < 0.05, **p < 0.01***p < 0.001, ****p < 0.0001. Error bars represent SEM.

To relate the distribution of CXCL12 to the possible requirement for CXCR4-CXCL12 interactions in DP thymocytes, we examined CXCR4 expression alongside a panel of maturational markers in WT mice. Initially, we examined CXCR4 in pre-selection DP (CD69^−^), post-selection DP (CD69^+^), immature SP4 and SP8 (CD69^+^CD62L^−^), and mature SP4 and SP8 (CD69^−^CD62L^+^) subsets (Fig. 2a). The expression of CXCR4 was also compared to that of CCR9, an additional chemokine receptor expressed by DP thymocytes. While both CXCR4 and CCR9 are highly and uniformly expressed by pre-selection DP thymocytes (Fig. 2b), compared to isotype control staining, we found that CXCR4 underwent distinct changes at subsequent stages of development. Thus, CXCR4 was still detectable in DP CD69^+^ thymocytes, but levels were significantly reduced compared to pre-selection DP CD69^−^ cells (Fig. 2b). Moreover, analysis of later stages of development provided evidence for continued CXCR4 downregulation, with expression levels on immature and mature SP4 and SP8 subsets only slightly higher that that seen with an isotype control (Fig. 2b). Interestingly, a different pattern of CCR9 expression was observed. Compared to isotype control staining, CCR9 expression was not downregulated between DP CD69^−^ and DP CD69^+^ thymocytes, with downregulation being initiated at the SP4 stage (Fig. 2b). Thus, detailed analysis of chemokine receptor expression during T-cell development indicates that pre-selection DP thymocytes undergo progressive and selective changes in CXCR4 during the initiation and completion of TCR-mediated thymic selection events. To examine this directly, we examined levels of CXCR4 and CCR9 in DP thymocytes from Nur77GFP mice, where GFP expression occurs following TCR signalling^44^. While CCR9 levels were comparable in pre-selection Nur77GFP^−^ DP and TCR-triggered Nur77GFP^+^ DP thymocytes, CXCR4 was significantly lower in the latter (Fig. 2c). Thus, CXCR4 downregulation correlates with TCR-mediated signalling in DP thymocytes.

To relate this intrathymic pattern of expression to events occurring post thymic egress, we compared expression of CXCR4 on the most mature CD69^−^CD62L^+^ SP thymocytes and subsets of splenic αβT-cells in Rag2pGFP mice, to allow direct analysis of Rag2GFP^+^ Recent Thymic Emigrants (RTE) and more mature GFP^−^ T-cells^45^. Compared to staining levels with an isotype control, and similar to CD69^−^CD62L^+^ mature SP thymocytes, CXCR4 levels on CD4^+^ and CD8^+^ RTE and CD62L^+^ naïve T-cells were very low, while a significant increase was observed between naïve T-cells and CD44^+^ memory T-cells (Fig. 2d). Thus, selective reduction of CXCR4 expression occurs during the earliest stages of DP thymocyte selection, resulting in very low levels of CXCR4 in SP thymocytes that are not altered in RTE and naïve T-cells. Interestingly however, transitional DP CD69^+^ thymocytes show intermediate levels of CXCR4 compared to their immature DP CD69^−^ counterparts and SP4 and SP8 progeny. Collectively, these findings indicate that CXCR4 has the potential to play a role in both pre-selection DP thymocytes, and those recently triggered by positive selection.

### Conventional αβT-Cells Develop Normally Following CXCR4 Deletion In DP Thymocytes

The alterations in CXCR4 expression that take place during positive selection of DP thymocytes, and the continued expression of CXCR4 in DP CD69^+^ intermediates led us to examine its role during and after positive selection. To this end, we crossed CD4^Cre^ mice^46^ with CXCR4^fl/fl^ mice^47^ to generate CD4^Cre^CXCR4^fl/fl^ (CXCR4 cKO) mice. In all of the experiments described here we used CD4^Cre^CXCR4^+/+^ mice as controls for CXCR4 cKO mice. Importantly, Cre expression in CD4^Cre^ mice has been shown previously not to have off-target effects, and to mirror endogenous CD4 gene expression, resulting in specific and effective gene deletion in DP thymocytes^48^ (Supplementary Figure 1), demonstrating the relevance of this model in analysing the role of CXCR4 in DP thymocytes.

**Figure 3:**
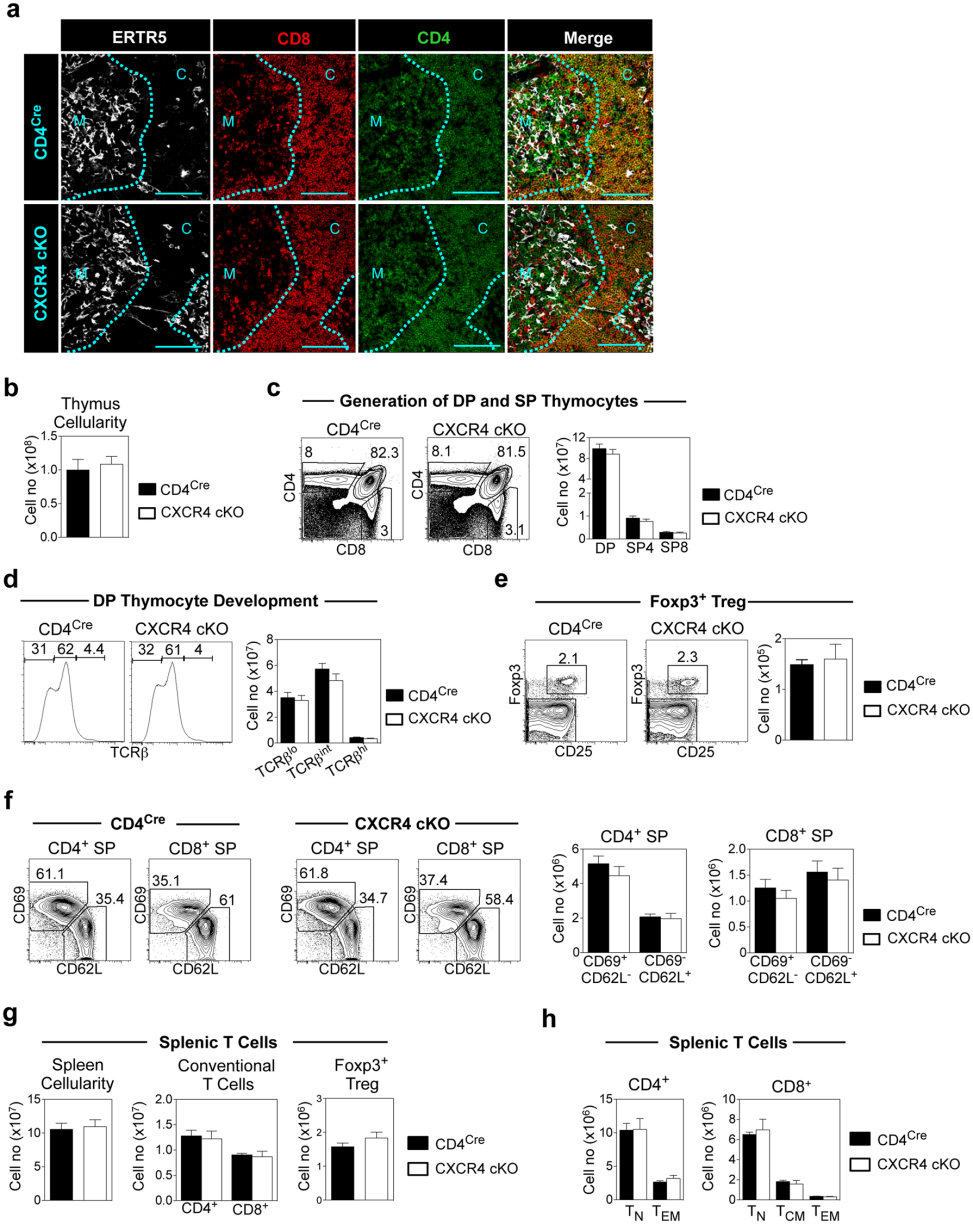
Conventional αβT-cell Development Occurs Normally In CXCR4 cKO Mice. (**a)** Immunofluorescence showing typical positioning of thymocytes in CD4^Cre^ and CXCR4 cKO mice, ‘C’ denotes cortex and ‘M’ denotes medulla, scale bar represents 100 μm. (**b)** Thymus cellularity of CXCR4 cKO compared to CD4^Cre^ mice. Representative FACS analysis and quantitation of **(c)** DP and SP thymocytes, **(d)** TCRβ^lo^, TCRβ^int^ and TCRβ^hi^ DP thymocytes, (**e)** CD25^+^Foxp3^+^ T-Reg, (**f)** CD69^+^CD62L^−^ immature and CD69^−^CD62L^+^ mature SP4 and SP8 thymocytes from CXCR4 cKO and CD4^Cre^ control thymus. (**g**) Spleen cellularity and numbers of CD4, CD8 and Foxp3^+^ T cells in the spleen. (**h**) Numbers of naïve (T_N_ CD62L^+^CD44^−^), central memory (T_CM_ CD62L^+^CD44^+^) and effector memory (CD62L^−^CD44^+^) CD4^+^ and CD8^+^ T cell subsets in the spleen of CD4^Cre^ and CXCR4 cKO mice. All quantitation is generated from at least three independent experiments where n > 7, unpaired Student’s *t* test shows no significant difference. Error bars represent SEM.

When we analysed the distribution of DP thymocytes in thymic sections from CXCR4 cKO mice by confocal microscopy, thymocytes were appropriately positioned in both control (CD4^Cre^) and CXCR4 cKO mice, with DP and SP thymocytes residing in the cortex and medulla respectively (Fig. 3a). Moreover, flow cytometric analysis revealed no differences in thymus cellularity (Fig. 3b) and similar numbers of DP, SP4 and SP8 thymocytes were seen (Fig. 3c). Indeed, when we used expression of TCRβ to subset DP thymocytes and identify cells preparing for (TCRβ^lo^), undergoing (TCRβ^int^), and emerging from (TCRβ^hi^) positive selection^49^, CD4^Cre^ control mice and CXCR4 cKO mice had equal numbers of DP thymocytes within each of these populations (Fig. 3d), suggesting that the DP thymocyte pool is not altered by lack of CXCR4 expression. Moreover, normal numbers of Foxp3^+^ Regulatory T-cells (Fig. 3e), immature (CD69^+^CD62L^−^), and mature (CD69^−^CD62L^+^) SP4 and SP8 thymocytes (Fig. 3f) were noted in CXCR4 cKO mice. Finally, and consistent with a normal programme of T-cell development and thymus emigration, we found that numbers of splenic CD4^+^ and CD8^+^ αβT-cells (Fig. 3h), and naïve and memory αβT-cell subsets^50^ (Fig. 3g) were comparable in CXCR4 cKO and CD4^Cre^ control mice. Together, these results show that CXCR4 expression by DP thymocytes is dispensable for their correct intrathymic positioning, and subsequent stages in conventional αβT-cell development.

### CXCR4 Expression By DP Thymocytes Is Dispensable For CD1d-Restricted iNKT-cell Development

**Figure 4:**
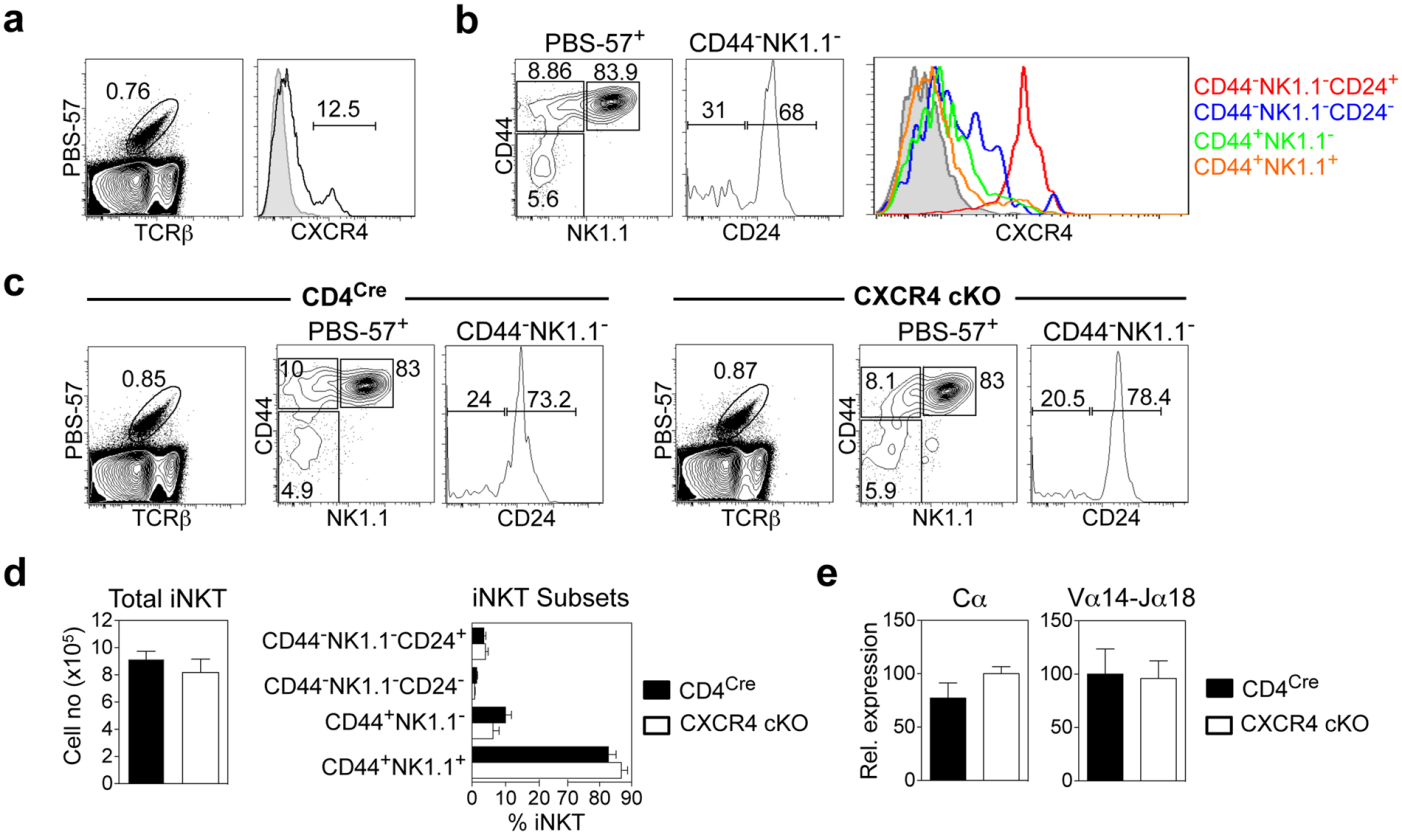
Intrathymic iNKT cell Development In CXCR4 cKO Mice. (**a**) Representative expression of CXCR4 by PBS-57^+^ CD1d-restricted thymocytes from WT mice. (**b**) Representative expression of CXCR4 by populations of PBS57^+^ thymic iNKT cells, defined as CD44^−^NK1.1^−^CD24^+^, CD44^−^NK1.1^−^CD24^−^, CD44^+^NK1.1^−^, and CD44^+^NK1.1^+^. Grey histograms show isotype control staining for CXCR4. (**c**) FACS analysis of total and iNKT cell subsets in control CD4^Cre^ and CXCR4 cKO mice. (**d)** Quantitation of total and iNKT cell subsets in control CD4^Cre^ and CXCR4 cKO mice. (**e)** TCR Vα14-Jα18 rearrangements in PBS-57^+^ depleted DP thymocytes from control or CXCR4 cKO mice. All quantitation is generated from at least three independent experiments where n > 7, unpaired Student’s *t* test shows no significant difference. Error bars represent SEM.

As well as acting as a substrate for conventional αβT-cells, pre-selection DP thymocytes also give rise to CD1d-restricted iNKT-cells via DP-DP thymocyte interactions in the cortex^51^. Interestingly, development of iNKT cells depends upon a normal DP thymocyte lifespan, which enables cells to undergo appropriate TCRα gene rearrangements to generate an invariant Vα14-Jα18 TCRα chain that forms part of the CD1d-reactive αβTCR^52–54^. To investigate whether CXCR4 plays a role in iNKT-cell development, we used CD1d tetramers loaded with the α-galactosylceramide analog PBS57 to monitor thymocyte populations for expression of CXCR4, and examined iNKT-cell development in CXCR4 cKO mice. Interestingly, a small proportion of CD1d-tetramer^+^ iNKT cells were CXCR4^+^ (Fig. 4a), and subdivision of the iNKT-cell population into subsets based on expression of CD44, NK1.1 and CD24^55^, revealed CXCR4 exclusively mapped to the most immature stage 0 cells (Fig. 4b). Interestingly, and despite expression of CXCR4 at early stages of iNKT-cell development, total numbers of thymic iNKT cells, and cells within NK1.1/CD44 subpopulations were comparable in control CD4^Cre^ and CXCR4 cKO mice (Fig. 4c,d). To further examine the requirement for CXCR4 during iNKT-cell development, we next measured Vα14-Jα18 TCR rearrangements in iNKT cell depleted DP thymocytes from CD4^Cre^ control and CXCR4 cKO mice by quantitative real time PCR^54^. Figure 4e shows that comparable levels of Vα14-Jα18 rearrangements were seen in control CD4^Cre^ and CXCR4 cKO mice. Collectively, our data show that CXCR4 expression in CD1d-restricted iNKT cells is limited to earliest intrathymic phases of their development, and that generation of iNKT-cells, including appropriate Vα14-Jα18 TCRα rearrangements, occurs independently of CXCR4 expression.

### CXCR4-Deficient DP Thymocytes Compete Effectively With WT Counterparts During Intrathymic Selection

**Figure 5:**
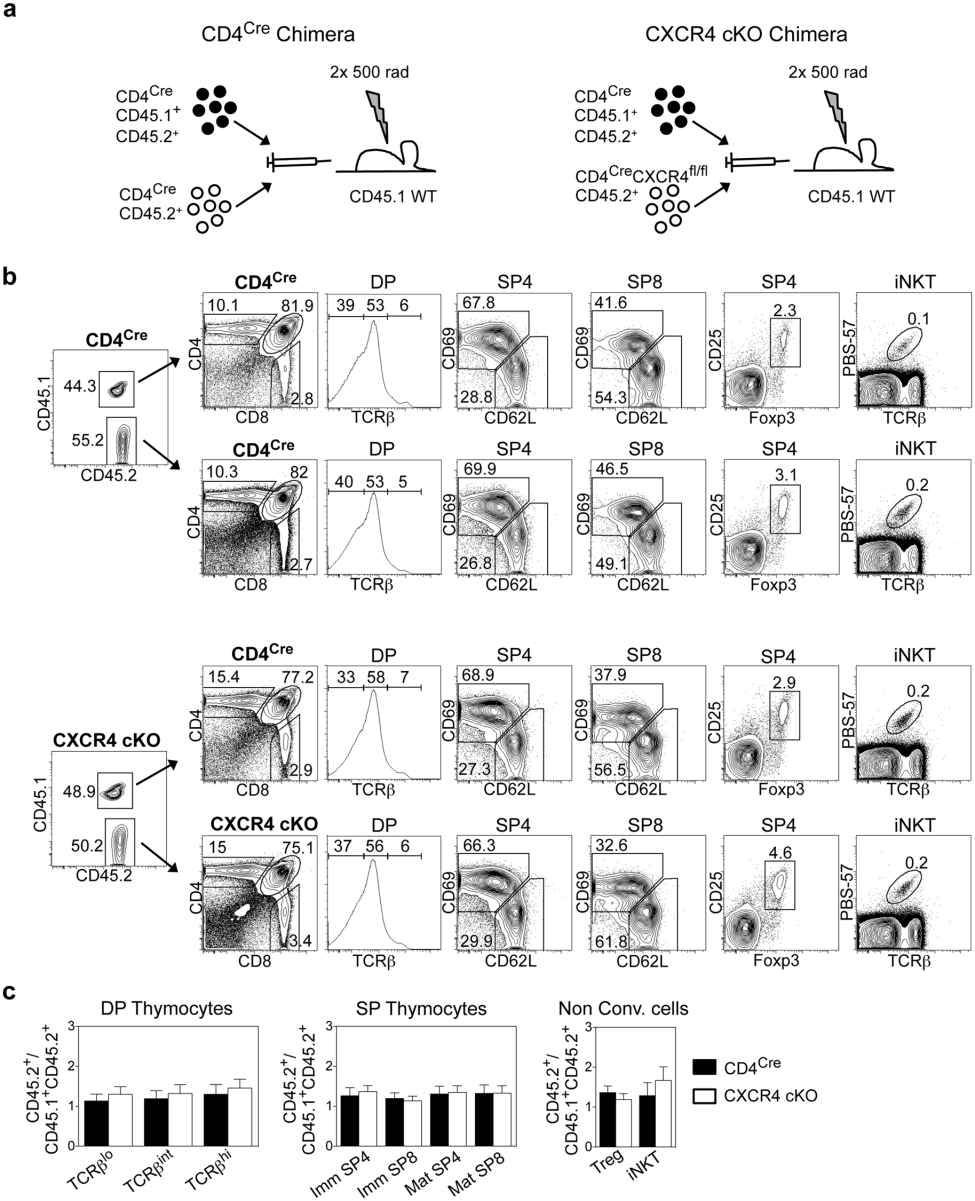
CXCR4 cKO Thymocytes Compete Effectively With WT Counterparts During T-cell Development. (**a)** Experimental schematic for the generation of competitive bone marrow chimeras. (**b**) FACS analysis of T-cell development within each donor population for CD4^Cre^ and CXCR4 cKO chimeras. (**c**) Ratio of CD45.2^+^/CD45.1^+^CD45.2^+^ cells in each type of chimera for the indicated populations of thymocytes. Data is normalised to chimerism at the ETP stage of development. Quantitation is generated from three independent experiments where n > 12, unpaired Student’s *t* test shows no significant difference. Error bars represent SEM.

In several cases, the importance of chemokine receptors for T-cell development is revealed only by mixed bone marrow (BM) chimeras, in which chemokine receptor deficient thymocytes operate under competitive conditions with WT counterparts^9, 12, 56^. Consequently, we examined whether a role for CXCR4 in T-cell development could be revealed in this setting. As described previously^56^, CD45.1^+^ hosts were irradiated and reconstituted with T-cell depleted BM from CD45.1^+^CD45.2^+^ CD4^Cre^ control mice, mixed at a 1:1 ratio with either CD45.2^+^ CD4^Cre^ control or CD45.2^+^ CD4^Cre^CXCR4^fl/fl^ BM cells (Fig. 5a). Mice were harvested after 8–10 weeks and thymic chimaerism determined by flow cytometry. The ratio of control CD4^Cre^:CXCR4 cKO cells was calculated and normalised to chimaerism seen within early thymic progenitors (Supplementary Figure 2) to account for any bias in chimaerism in stages prior to CD4^Cre^-mediated deletion. Interestingly, detailed flow cytometric analysis of stages in both conventional and CD1d-restricted αβT-cell development showed that CXCR4 cKO thymocytes underwent a normal programme of intrathymic development alongside their CD4^Cre^ control counterparts (Fig. 5b,c). Thus, CXCR4 cKO DP thymocytes are effective competitors with CD4^Cre^ control thymocytes in their ability to complete intrathymic conventional and CD1d-restricted αβT-cell development.

## Discussion

During the intrathymic development of αβT-cells, DP thymocytes that reside in the cortex undergo thymic selection events determined by the specificities of the αβTCR they express. While conventional αβT-cells are selected via recognition of self-peptide/MHC molecules expressed by cTEC, DP-DP thymocyte interactions in the thymic cortex can result in the generation of CD1d-restricted iNKT-cells. Thus, the thymic cortex represents an important microenvironment for the intrathymic generation of both innate and conventional αβT-cells. However, the specialisation of cTEC that enables them to control these processes, as well as the cell surface counter receptors expressed by thymocytes that are required for appropriate cTEC-DP thymocytes interactions, are poorly understood. Here, we have examined the role of CXCR4-CXCL12 interactions during DP thymocyte development, including differentiation along conventional and CD1d-restricted αβT-cell lineages. Our initial examination of the intrathymic distribution of CXCL12 using CXCL12^dsRed^ reporter mice indicates that this marker is widespread throughout the thymic cortex, and within TEC populations it is selectively expressed by the majority of cTEC. Interestingly, this pattern of expression differs to that previously reported for the intrathymic production of CXCL12. For example, while some studies demonstrated *Cxcl12* mRNA expression to be restricted to the subcapsular region of the neonate^42^, others reported rare *Cxcl12* mRNA^+^ cytokeratin-expressing cells within the cortex of the adult^25^. Moreover, other reports described CXCL12 protein predominantly in the adult thymus medulla^20^ or in both the cortex and medulla^28^. The reasons for these discrepancies are not fully clear. However, while they may likely reflect different methods to measure CXCL12 expression, our analysis of dsRed expression in cTEC matches well with *Cxcl*12 mRNA levels in these cells. In addition, it is important to note that the fluorescent protein distribution described here may not represent CXCL12 protein production throughout the cortex. However, our study does provide evidence that the CXCL12 gene locus in active in the majority of cTEC, suggesting that most if not all DP thymocytes may be located close to a source of this chemokine. Moreover, this expression pattern in CXCL12^dsRed^ mice indicates this mouse strain may also represent a useful tool in future studies to analyse and isolate cells of the cTEC lineage by flow cytometry and/or confocal microscopy. In addition, by measuring CXCR4 expression and relating this to early stages in the maturation of both MHC-restricted and CD1d-restricted αβT-cells, we show that CXCR4 downregulation initially occurs following the onset of thymic selection, and differing levels of CXCR4 identify DP thymocytes at pre- and post-stages of positive selection initiation. Thus, changes in CXCR4 within DP thymocytes can serve as a useful means to identify cells undergoing thymic selection.

We also found this pattern of CXCR4 to be selective, in that DP thymocytes retained CCR9 expression until the SP stage of development. Interestingly, this continued post-selection expression of CCR9 is antagonised by interactions between plexinD1 and semaphorin 3E, which repress responsiveness to CCL25^57^. Thus, while additional receptors may influence thymocyte migration and positioning controlled by CCR9, the pattern of expression for CXCR4 suggests it may directly regulate the intrathymic positioning of thymocytes. Consistent with this, experiments monitoring the positioning of human thymocytes in mouse thymic slices *in vitro* suggested CXCR4 as a cortex-retention factor for pre-selection thymocytes^26^. Importantly however, the generation and analysis of T-cell development in CXCR4 cKO mice described here, in which CD4^Cre^ results in uniform CXCR4 deletion by the DP stage, demonstrates that CXCR4 is dispensable for *in vivo* intrathymic DP positioning and downstream αβT-cell development. Importantly, and consistent with this, efficient αβT-cell development in CXCR4 cKO mice was not altered under competitive conditions with control cells. Interestingly, we also found normal numbers of peripheral CD4^+^ and CD8^+^ αβT-cells in cCXCR4 cKO mice, indicating that CXCR4 is not required for the exit of mature cells from the thymus. This finding has significance, given previous studies using fetal thymus organ cultures and the CXCR4 antagonist AMD3100 suggest a role for CXCR4 in both thymocyte retention and egress^29, 32, 58^. However, it is important to note that the lack of a thymic emigration defect in CXCR4 cKO mice reported here fits well with the absence of an intrathymic accumulation of mature SP thymocytes that accompanies egress blockade^59^. In summary, examination of the intrathymic distribution of CXCR4 and CXCL12 alongside the generation and analysis of CXCR4 cKO mice demonstrates that CXCR4 expression in DP thymocytes is dispensable for downstream αβT-cell development. This indicates that the role of CXCR4 in thymocyte development is stage-specific and limited to earlier events that include thymus colonization and pre-TCR mediated differentiation.

## Methods

### Mice

The following mice on a C57BL/6 background were used at 8–12 weeks of age: Wild-type (WT) C57BL/6 and congenic CD45.1^+^ BoyJ mice, Nur77^GFP^ ^44^ and Rag2GFP^45^. CXCL12^dsRed^ ^43^ CD4^Cre^ ^46^ and CXCR4^fl/fl^ ^47^ mice were obtained from Jackson Laboratory. CD4^Cre^CXCR4^fl/fl^ (CXCR4 cKO) mice were generated by crossing CD4^Cre^ mice to CXCR4^fl/fl^ mice. In all experiments, CD4^Cre^CXCR4^+/+^ mice were used as controls for CXCR4 cKO mice. In bone marrow chimera experiments, CD4^Cre^ control mice carried the congenic markers CD45.1 and CD45.2. Husbandry, housing and experimental methods involving mice were performed at the Biomedical Services Unit at the University of Birmingham in accordance the Local Ethical Review panel and national Home Office regulations (animal project licence number PPL30/2929).

### Antibodies and Flow Cytometry

For analysis of thymocytes, splenocytes and iNKT cells, tissues were mechanically disrupted to achieve a single cell suspension. Cells were stained with Abs specific for CD4 (RM4-5), CD8α (53-6.7), CD69 (H1.2F3), CD62L (MEL-14), CXCR4 (2B11), CCR9 (CW-1.2), TCRβ (H57-597), CD25 (PC61.5), CD44 (IM7), NK1.1 (PK136), CD24 (M1/69), CD45.1 (A20), CD45.2 (104), Foxp3 (FJK-16s). To detect iNKT-cells, we used a tetramer (NIH Tetramer Facility), which consists of CD1d loaded with PBS57, an analogue of α-galactosylceramide. Foxp3 staining was performed using an intracellular Foxp3 kit purchased from eBioscience. Streptavidin-BV786 or APC was used to reveal staining with biotinylated antibodies. For the analysis of stromal cells^60^ thymi were enzymatically digested using collagenase dispase (2.5 mg/ml; Roche) and DNase 1 (40 mg/ml; Roche). Prior to surface staining, CD45^+^ cells were depleted using anti-CD45 microbeads and LS columns (Miltenyi Biotec). Cells were stained with the lectin Ulex Europaeus Agglutinin-1, and antibodies specific for CD45 (30-F11), EpCAM-1 (G8.8), Ly51 (6C3), Podoplanin (8.1.1), CD31 (390), TER-119 (TER-119). Antibodies were conjugated to Brilliant Violet (BV) 711, BV785, BV510, Pacific Blue, eFluor 450, PE, PE-Cy7, PerCP–eFluor 710, APC, APC–eFluor 780, Alexa Fluor 700 and biotin, and were purchased from eBioscience, BD Biosciences, BioLegend, or Vector Laboratories. Data were acquired using a BD LSRFortessa and were analyzed using FlowJo software (Tree Star). Forward and side scatter gates were set to exclude nonviable and aggregated cells.

### Quantitative Real-time PCR

Expression of *Cxcl12* mRNA by thymic stromal cell subsets was determined as previously described^60^. Thymi were enzymatically digested using collagenase dispase (2.5 mg/ml; Roche) and DNase 1 (40 μg/ml; Roche), followed by CD45 depletion using anti-CD45 microbeads (Miltenyi Biotec). Cells were stained with the lectin Ulex Europaeus Agglutinin 1 (UEA-1) and antibodies to CD45 (30-F11), EpCAM1 (G8.8), Ly51 (6C3), TER-119 (TER-119), podoplanin (8.1.1). CD45^−^EpCAM1^+^UEA-1^−^Ly51^+^ cortical thymic epithelial cells (cTEC) and CD45^−^EpCAM1^−^podoplanin^+^ mesenchyme were FACS sorted using a MoFlo XDP (Beckman Coulter). CD31^+^ endothelial cells were sorted using anti-CD31 microbeads (Miltenyi Biotec) and MS columns, according to the manufacturer’s instructions. Vα14-Jα18 TCRα gene rearrangements were analysed exactly as described^54^. Briefly, PBS-57^−^CD4^+^CD8^+^ thymocytes were sorted from control and CXCR4 cKO mice using a MoFlo XDP (Beckman Coulter). Sorted populations were analyzed by quantitative PCR (qPCR) for expression of the indicated genes^19^. Primers used are as follows: β-actin (QuantiTect Mm_Actb_1_SG primer assay Qiagen QT00095242) and primer sequences for the target genes (https://www.ncbi.nlm.nih.gov/pmc/articles/PMC4458460/):

Cα forward: 5′-CCTCTGCCTGTTCACCGACTT-3′ and reverse: 5′-TGGCGTTGGTCTCTTTGAAG-3′

Vα14-Jα18 forward: 5′-GTGTCCCTGACAGTCCTGGT-3′ and reverse: 5′-CAAAATGCAGCCTCCCTAAG-3′

Cxcl12 forward: 5′-GCTCTGCATCAGTGACGGTA-3′ and reverse: 5′-TGTCTGTTGTTGTTCTTCAGC-3′

Levels of mRNA were normalized to β-actin; relative expression represents the mean (±SEM) of replicate reactions, and data is representative of at least 2 independently sorted biological samples.

### Confocal Microscopy

Thymic tissue from CXCL12^dsRed^ mice was treated for 2 hours with 2% PFA followed by overnight treatment with 18% sucrose prior to snap freezing. Freshly isolated thymic tissue from non-fluorescent reporter mice was mounted in OCT and snap frozen in liquid nitrogen. 8μm cryosections were prepared, air-dried and fixed for 20 minutes in cold acetone. Antibodies used for immunolabelling of tissue sections were: CD4 Alexa Fluor 647 (RM4-5, BioLegend), CD8 Biotin (53-6.7, eBioscience), CD205 Alexa Fluor 488 (205yekta, eBioscience), Keratin-5 (Poly19055, Covance) ERTR5^61^, Streptavidin Alexa Fluor 555 (Thermofisher), goat anti-rat Alexa Fluor 488 (Thermofisher) All sections were counterstained with DAPI (4′, 6-diamidino-2-phenylindole, Thermofisher). Images were acquired using a Zeiss LSM 780 confocal, or Zeiss AxioScan Slide Scanner, in conjunction with Zeiss Zen Black software.

### Generation of Bone Marrow Chimeras

Bone marrow was extracted from adult CD4^Cre^ (CD45.1^+^CD45.2^+^), CD4^Cre^ (CD45.2^+^), and CD4^Cre^CXCR4^fl/fl^ (CD45.2^+^) mice. T-cells were depleted using anti CD3-PE and anti-PE microbeads (Miltenyi Biotec) according to the manufacturer’s instructions. CD45.1^+^ BoyJ mice were lethally irradiated (2 × 500 rad) and reconstituted intravenously with 5 × 10^6^ BM cells mixed at a 1:1 ratio to form two sets of chimeras with congenically marked donor cells (CD4^Cre^:CD4^Cre^ and CD4^Cre^:CD4^Cre^CXCR4^fl/fl^). Chimaeric mice were harvested 8–10 weeks after reconstitution, and the contribution of CD45.1^−^CD45.2^+^ cells to each chimeric mouse assessed. Analysis was normalised to the chimaerism within ETP, defined as lin^−^CD44^+^CD25^−^CD117^+^. The following lineage markers were used: CD3ε (145-2C11), CD4 (GK1.5), CD8α (53-6.7), CD8β (H35-17.2), CD11b (M1/70), CD11c (N418), B220 (RA36B2), Ly-6G (RB6-865), NK1.1 (PK136), Ter-119 (TER-119), TCRβ (H57-597), and TCRδ (GL3).

## Electronic supplementary material

Supplementary Figures
