# Supplementary material for: Progressive Changes in CXCR4 Expression That Define Thymocyte Positive Selection Are Dispensable For Both Innate and Conventional αβT-cell Development
Source: Sci Rep. 2017 Jul 11;7:5068. doi: 10.1038/s41598-017-05182-7 (PMC5505955; doi:10.1038/s41598-017-05182-7)
Supplement: Supplementary file 1 — Supplementary Figures [file 41598_2017_5182_MOESM1_ESM.pdf]

Progressive Changes in CXCR4 Expression That Define Thymocyte Positive Selection Are Dispensable For Both Innate and Conventional αβT-cell Development

Beth Lucas, Andrea J. White, Sonia M. Parnell, Peter M. Henley, William E. Jenkinson and Graham Anderson*

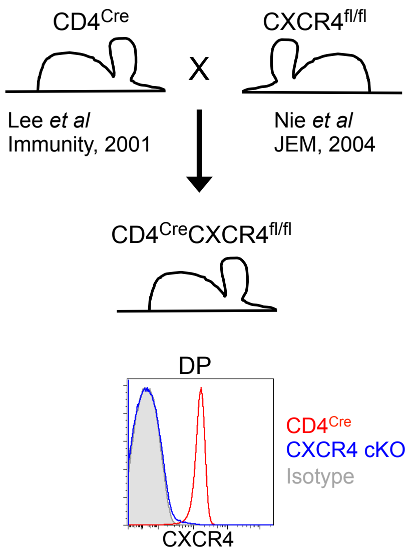

CD4^{Cre} Mediated Deletion of CXCR4 in DP Thymocytes

CD4^{Cre} and CXCR4$^{fl/fl}$ mice were used to generate CD4^{Cre}/CXCR4$^{fl/fl}$ (CXCR4 cKO) mice. DP thymocytes from CD4Cre (red line) and CXCR4 cKO (blue line) were stained with anti-CXCR4 antibodies. The grey histogram shows levels of expression using an isotype control antibody.

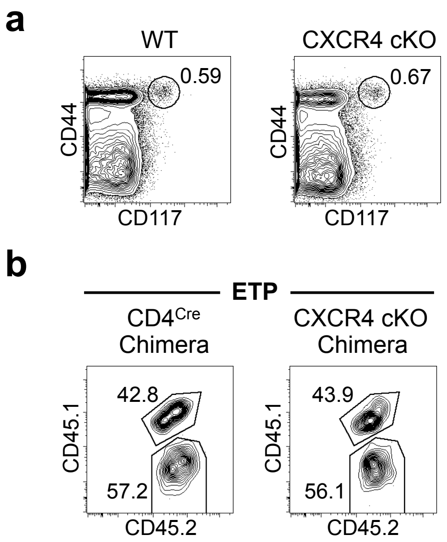

Quantitation of ETP For Analysis of Mixed BM Chimaeras

(a) Shows the proportion of Lin^- ($CD4^-CD8^-CD3^-CD25^-CD11b^-CD11c^-TER119^-NK1.1^-TCR\beta^-TCR\delta^-GR1^-$) $CD44^+CD117^+$ early thymic progenitors in $CD4^{Cre}$ and CXCR4 cKO mice. (b) shows ETP populations in control ($CD4^{Cre}$:$CD4^{Cre}$) and CXCR4 cKO ($CD4^{Cre}$: CXCR4 CKO) chimaeras, identified by expression of CD45.1 and CD45.2 Note that ETP populations are equivalent in both types of chimaeras.
